# Intestinal Innate Antiviral Immunity and Immunobiotics: Beneficial Effects against Rotavirus Infection

**DOI:** 10.3389/fimmu.2016.00563

**Published:** 2016-12-05

**Authors:** Julio Villena, Maria Guadalupe Vizoso-Pinto, Haruki Kitazawa

**Affiliations:** ^1^Immunobiotics Research Group, Tucuman, Argentina; ^2^Laboratory of Immunobiotechnology, Reference Centre for Lactobacilli (CERELA-CONICET), Tucuman, Argentina; ^3^Food and Feed Immunology Group, Laboratory of Animal Products Chemistry, Graduate School of Agricultural Science, Tohoku University, Sendai, Japan; ^4^Faculty of Medicine, INSIBIO (UNT-CONICET), National University of Tucuman, Tucuman, Argentina; ^5^Livestock Immunology Unit, International Education and Research Center for Food and Agricultural Immunology (CFAI), Graduate School of Agricultural Science, Tohoku University, Sendai, Japan

**Keywords:** immunobiotics, rotavirus, inflammation, TLR3, intestinal epithelial cells, intraepithelial lymphocytes

## Abstract

The mucosal tissues of the gastrointestinal tract are the main portal entry of pathogens such as rotavirus (RV), which is a leading cause of death due to diarrhea among young children across the globe and a major cause of severe acute intestinal infection in livestock animals. The interactions between intestinal epithelial cells (IECs) and immune cells with RVs have been studied for several years, and now, it is known that the innate immune responses triggered by this virus can have both beneficial and detrimental effects for the host. It was demonstrated that natural RV infection in infants and experimental challenges in mice result in the intestinal activation of pattern recognition receptors (PRRs) such as toll-like receptor 3 (TLR3) and striking secretion of proinflammatory mediators that can lead to increased local tissue damage and immunopathology. Therefore, modulating desregulated intestinal immune responses triggered by PRRs activation are a significant promise for reducing the burden of RV diseases. The ability of immunoregulatory probiotic microorganisms (immunobiotics) to protect against intestinal infections, such as those caused by RVs, is among the oldest effects studied for these important group of beneficial microbes. In this review, we provide an update of the current status on the modulation of intestinal antiviral innate immunity by immunobiotics and their beneficial impact on RV infection. In addition, we describe the research of our group that demonstrated the capacity of immunobiotic strains to beneficially modulated TLR3-triggered immune response in IECs, reduce the disruption of intestinal homeostasis caused by intraepithelial lymphocytes, and improve the resistance to RV infections.

## Introduction

One of the leading causes of children mortality is preventable infectious diseases (1, 2). Rotavirus (RVs), calicivirus, astrovirus, and adenovirus account to the viral etiologic agents of gastroenteritis in humans (3, 4). RV, a naked double-strand RNA (dsRNA) virus, is the most common cause of severe dehydrating diarrhea in children (5, 6). The main symptoms of RVs gastroenteritis are nausea, low-grade fever, vomit, and acute watery diarrhea. Even though two oral vaccines containing attenuated live viruses are being used globally, Rotarix (GlaxoSmithKline) and RotaTeq (Merck), the epidemic in the developing world is far from being controlled (6, 7). Vaccine effectiveness is reduced in developing areas, and some possible reasons are children infected at an early age, high viral challenge loads, and the lack of transferred maternal antibodies (8, 9).

Some lactic acid bacteria (LAB) strains are able to impact on human and animal health by modulating the mucosal and systemic immune systems. Those immunoregulatory probiotic LAB, known as immunobiotics, provide protection against viral infections by modulating innate and adaptive antiviral immunity. Thus, several reports have shown that immunobiotic LAB shorten the duration of diarrhea, reduce the number of episodes, diminish RVs shedding, normalize gut permeability, and increase the production of RVs-specific antibodies (10–12).

The purpose of this review is to provide an update of the current status on the modulation of intestinal antiviral innate immunity by immunobiotics, and their beneficial impact on RVs infection. We also highlight some results of our group, which demonstrate the capacity of immunobiotic strains to beneficially modulate toll-like receptor (TLR)-3-triggered immune response in intestinal epithelial cells (IECs), reduce the disruption of intestinal homeostasis caused by intraepithelial lymphocytes (IELs), and improve the resistance to RVs infection.

## Intestinal Antiviral Innate Immune Response and Rotavirus

**Figure 1:**
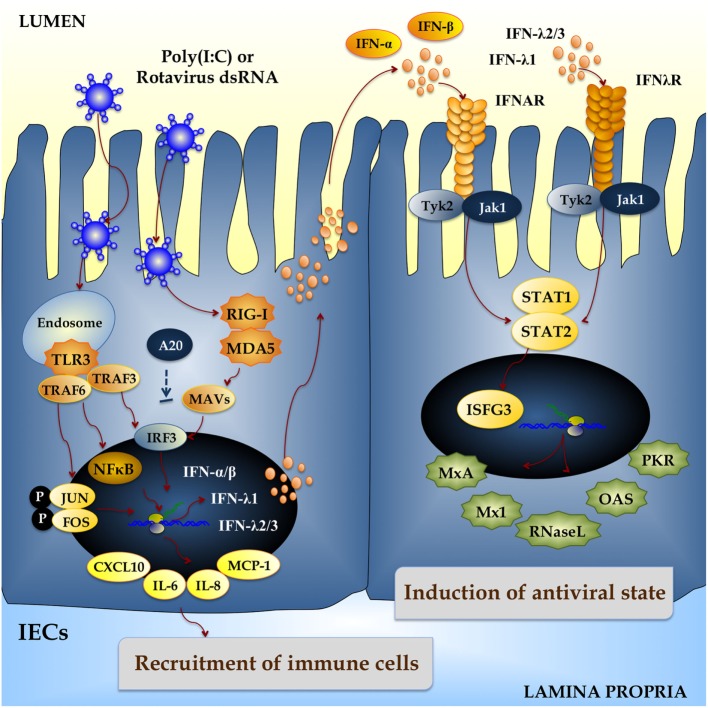
**Innate immune response against rotavirus in intestinal epithelial cells (IECs)**. Rotavirus double-strand genomic RNA activates toll-like receptor 3 (TLR3), retinoic acid-inducible gene-I (RIG-I), and melanoma differentiation-associated gene-5 (MDA-5), which are pattern recognition receptors (PRRs) expressed in IECs. Cellular signaling cascades are activated and converge at the level of interferon (IFN) regulatory factor-3 (IRF3) that upregulate the expression of type I (IFN-α, IFN-β) and type III (IFNλ1, IFNλ2/3) IFN, which in turn induces the synthesis of IFN-stimulated genes with antiviral activities (MxA, Mx1, RNase L, OAS, PKR). Antiviral PRRs also activate nuclear factor κB (NF-κB) pathway and induce the secretion of proinflammatory cytokines and chemokines (IL-6, IL-8, MCP-1, CXCL10). Those effects could be imitated *in vitro* and *in vivo* by administration of the dsRNA synthetic analog poly(I:C).

**Figure 2:**
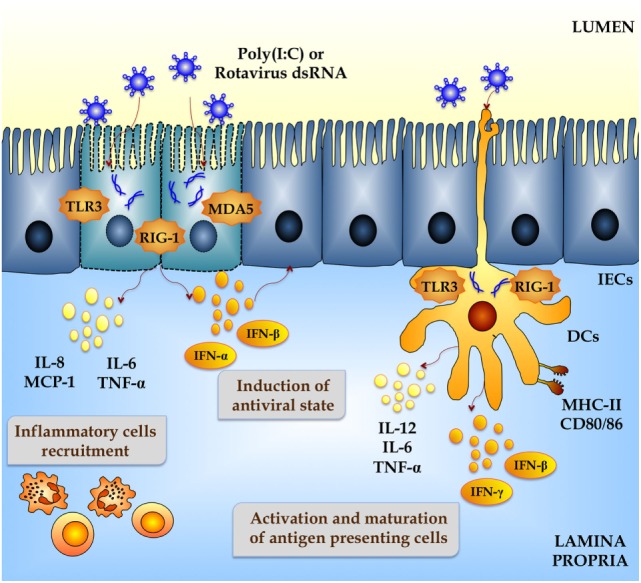
**Innate immune response against rotavirus in intestinal mucosa**. Rotavirus double-strand genomic RNA activate toll-like receptor 3 (TLR3), retinoic acid-inducible gene-I (RIG-I), and melanoma differentiation-associated gene-5 (MDA-5), which are pattern recognition receptors (PRRs) expressed in intestinal epithelial cells (IECs) and dendritic cells (DCs). Activation of antiviral PRRs in the intestinal mucosa increases the production of type I IFN (IFN-α, IFN-β), IFN-γ, and proinflammatory cytokines and chemokines (TNF-α, IL-6, IL-8, IL-12, MCP-1), which improves the antiviral state in IECs, induces the recruitment and activation of immune cells and the maturation of DCs. Those effects could be imitated *in vitro* and *in vivo* by administration of the dsRNA synthetic analog poly(I:C).

Upon RVs internalization, the capsid uncoats loosing VP4 and VP7, the outer surface proteins, and yielding a transcriptionally active double-layered particle. The eleven segments of dsRNA viral genome are transcribed directing the synthesis of structural and non-structural proteins and serving as templates for the complementary strand of genomic RNA (13). The IEC senses viral dsRNA through pattern recognition receptors (PRRs), such as TLR3, retinoic acid-inducible gene-I (RIG-I), and melanoma differentiation-associated gene-5 (MDA-5), and cellular signaling cascades are activated to react to viral infection (14–16) (Figure 1). One of the major innate responses against dsRNA viruses relies on the activation of those PRRs, which leads to the production of cytokines and chemokines by IECs and immune cells. Thus, RVs dsRNA triggers the production of IL-8, IP-10, IL-6, TNF-α, and IL-15 in IECs *via* the TLR3-, RIG-I-, and MDA5-activated pathways inducing recruitment and activation of macrophages and NK cells and stimulating adaptive B- and T-cell immune responses. As a result of PRRs activation, interferons (IFNs) and IFN-regulated gene products are also produced and they play a key role in establishing an antiviral state for virus clearance and restriction of spread (Figure 1). Type I and III IFNs limit RV infection *in vitro*, and their levels are augmented in RVs-infected children and animals (17–19). Both families of IFN are immediately produced upon RV infection, elicit responses on different types of receptors, and temporally and spatially regulated in the gastrointestinal tract (20). Another evidence suggesting that IFNs are crucial to limit RV infection relies on the fact that this virus has evolved mechanisms to manipulate IFNs signaling such as the type I IFNs damping NSP1 protein (21). While TLR3 mainly recognizes viral components such as viral nucleic acid in endosomal compartments, RIG-I and MDA-5 recognize cytoplasmatic dsRNA. These pathways converge at the level of IFN regulatory factor-3 (IRF3) (18, 22, 23). Upon IRF3 phosphorylation, antiviral responses initiate the activation of type I IFN, which in turn induces the synthesis of interferon-stimulated genes (ISGs), secretion of proinflammatory cytokines, and activation and maturation of antigen-presenting cells (APCs) (Figure 2).

**Figure 3:**
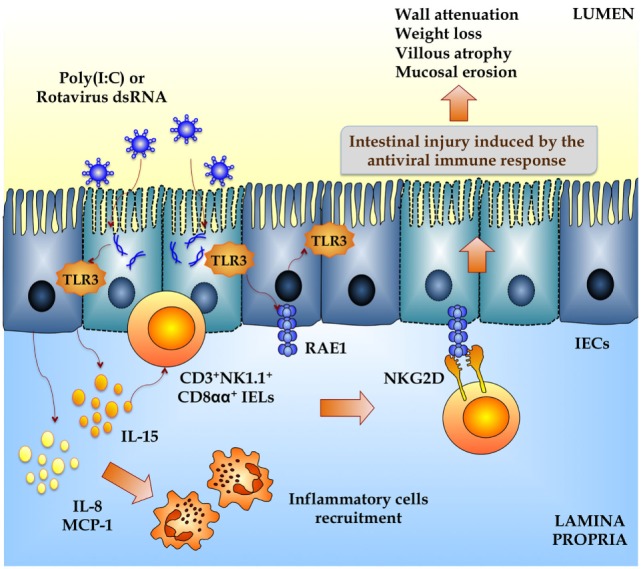
**Inflammatory damage of the intestinal mucosa induced by rotavirus in a toll-like receptor 3-deppendent manner**. Both purified rotavirus double-strand genomic RNA and poly(I:C) induce severe mucosal intestinal damage *via* TLR3 activation and intestinal epithelial cells (IECs) and intraepithelial lymphocytes (IELs) interaction. Activation of TLR3 in IECs increases the expression of proinflammatory cytokines and chemokines (MCP-1, IL-8, IL-15) and retinoic acid early inducible-1 (RAE1). IL-15 produced by IECs induce the recruitment of CD3^+^NK1.1^+^CD8αα^+^ IELs, which mediates epithelial destruction and mucosal injury by the NKG2D receptor expressed on these cells that is able to recognize RAE1. This intestinal TLR3-IECs-IELs interaction induces villous atrophy, mucosal erosion, and gut wall attenuation.

Poly(I:C), a synthetic analog of dsRNA, when administered intraperitoneally to mice mimics the local intestinal immune response elicited by an enteric viral infection (24, 25). Both purified RVs dsRNA and poly(I:C) are able to induce severe mucosal damage in the gut *via* TLR3 activation including villous atrophy, mucosal erosion, and gut wall attenuation (24). IELs, which are mostly T cells distributed as single cells within the epithelial cell layer, play a critical role in disrupting epithelial homeostasis caused by abnormal TLR3 signaling (Figure 3) (24). Due to their key location at the interface between the inner intestinal tissue and the lumen, these specialized immune cells are important as a first line of defense against microbes and in maintaining the epithelial barrier homeostasis. The majority of IELs are CD8^+^ being simply classified as CD8αα^+^ or CD8αβ^+^. The CD8αβ^+^ IELs bear the hallmarks of adaptive immune cells, whereas the CD8αα^+^ IELs are considered innate immune cells (26). When TLR3 is abnormally activated by poly(I:C) and RVs, genomic dsRNA, IL-15, and CD3^+^NK1.1^+^CD8αα^+^ IELs are involved in the disruption of epithelial homeostasis. In addition, it was demonstrated that TLR3 activation in IECs induces the expression of retinoic acid early inducible-1 (RAE1), which mediates epithelial destruction and mucosal injury by interacting with the NKG2D receptor expressed on IELs (27) (Figure 3).

Thus, increasing our understanding of how PRRs such as TLR3 are activated and regulated in immune cells and IECs may help designing effective therapies for the prevention and/or treatment of viral diseases.

## Beneficial Effects of Immunobiotics in Rotavirus Infection

Several studies have demonstrated that immunobiotics are able to improve the outcome of RV infection in human and livestock animals.

### Effects of Immunobiotics in Humans

*Lactobacillus rhamnosus* GG is probably the most studied probiotic bacteria in the context of intestinal viral infections. Isolauri et al. (28) first described for more than 20 years, a protective effect of *L. rhamnosus* GG strain in RVs gastroenteritis in infants and children. In this study, the patients who received either a *L. rhamnosus* GG-fermented milk product or a *L. rhamnosus* GG freeze-dried powder after oral rehydration presented a significantly shorter duration of diarrhea when compared to the placebo group. Later, Majamaa et al. (29) conducted a study, in which 6- to 35-month-old children with RVs gastroenteritis received either *L. rhamnosus* GG, *Lactobacillus acidophilus* or a combination of *Streptococcus thermophilus* with *L. delbrückii* subsp. *bulgaricus* twice daily for 5 days. Only children who received *L. rhamnosus* GG had shorter diarrhea duration. The protective effect was related to augmented intestinal and serum IgA concentration, and a higher number of specific antibody-secreting cells to RVs. Additional studies showed that the consumption of *L. rhamnosus* GG is able to shorten the diarrheal phase in children suffering from RVs infection, an effect that was associated with increased concentrations of IgA antibodies as well (28–33). Furthermore, meta-analysis showed that the administration of *L. rhamnosus* GG to hospitalized children reduced the overall incidence of health care-associated diarrhea, including symptomatic RVs gastroenteritis (34). In spite of this evidence, it is important to notice that the shortening of diarrhea already at day 3 after probiotic treatment strongly suggests that the main therapeutic effect involves innate immune responses rather than the modulation of adaptive immunity (35).

Another example of probiotic treatment for alleviating RVs gastroenteritis was the use of *Lactobacillus reuteri*, which has been daily administered to hospitalized children with acute diarrhea for the length of hospitalization (up to 5 days). *L. reuteri* shortened both the duration of RVs diarrhea and the disease severity, when compared to the placebo group (36). *L. sporogenes* daily administered to newborns during 1 year, prevented the incidence and also diminished the duration of acute RVs diarrhea (37). Fang et al. (38) demonstrated that a minimal effective dose of *L. rhamnosus* significantly reduced fecal shedding RVs concentration in pediatric patients. Although the administration of lyophilized *Lactobacillus paracasei* strain ST11 daily for 5 days had a clinically significant benefit in the management of non-RVs-induced diarrhea, ST11 treatment against severe RVs diarrhea was ineffective (39). Children with RV infection who received milk-based formula supplemented with either *B. animalis* Bb12 alone or combined with *S. thermophilus* had fewer RVs infections (40).

In Argentina, mucosal infections such as bronchitis and diarrhea are the most common infectious diseases in children (41–43). In a randomized controlled trial conducted by Villena et al. (44), *L. rhamnosus* CRL1505 (administered in a yogurt formulation) improved mucosal immunity and reduced the incidence and severity of intestinal and respiratory infection in children. Hence, the incidence of infectious events was reduced from 66% in the placebo group to 34% in the group that received the probiotic yogurt. Furthermore, there was also a significant reduction in the occurrence of indicators of disease severity such as fever and the need for antibiotic treatment in children receiving the probiotic yogurt (44). Therefore, the results of this trial suggested that consumption of yogurt containing *L. rhamnosus* CRL1505 was helpful to reduce the burden of common childhood morbidities, especially those associated to viral infections including RVs (44).

### Effects of Immunobiotics in Livestock Animals

Apart from the beneficial effects of immunobiotics on humans, some studies have evaluated their antiviral and anti-inflammatory activities in animals. Zhang et al. (45) reported that probiotic administration to gnotobiotic pigs challenged with RVs did not yielded differences in virus titers with respect to the placebo group. Nonetheless, LAB administration downregulated the recruitment of viral-activated monocytes/macrophages into the intestinal tract thereby limiting the inflammation induced by the virus (45).

In another study, it was shown that systemic monocyte/macrophage and APCs responses were modulated by immunobiotics in the context of a RV infection (45). Probiotic LAB induced strong TLR2-expressing APCs responses in blood and spleen, RVs induced a TLR3 response in spleen, and TLR9 responses were induced by RVs (as measured in immune cells isolated from spleen) and LAB (as determined in blood circulating immune cells). Immunobiotics and RVs had an additive effect on TLR2- and TLR9-expressing APCs responses, consistent with the adjuvant effect of LAB. Immunobiotics augmented IFN-γ and IL-4 levels in serum, but suppressed TLR3- and TLR9-expressing APCs responses in spleen and the serum IFN-α response induced by virulent RVs (46).

During RVs infections in weaned pigs, there is evidence of disruption of the barrier function as evidenced by the decreased villus height and crypt depth, lower levels of IgA, IL-4, and mucin 1 as well a reduced transcription of ZO-1, occludin, and Bcl-2 in jejunal mucosa (47). Some of these effects have been partially associated with alterations of transforming growth factor (TGF)-β production (48). Azevedo et al. (48) demonstrated that immunobiotic LAB further enhanced the Th1 and Th2 cytokine responses to RV infection as indicated by significantly higher concentrations of IL-12, IFN-γ, IL-4, and IL-10 in RVs-infected gnotobiotic pigs. LAB also helped to maintain immunological homeostasis during RV infection by regulating TGF-β production. It was also shown that treatment of pigs with *L. rhamnosus* GG modulated TGF-β and promoted the enhancement of intestinal epithelial tight junctions, which may contribute to the preservation and restoration of the gut homeostasis after RV infection (11). Further evidence was reported by Maragkoudakis et al. (12) demonstrating that *Lactobacillus casei* Shirota and *L. rhamnosus* GG protected porcine and goat epithelial cells from RVs and other transmissible gastroenteritis viruses.

## Cellular and Molecular Mechanisms of Immunobiotics Actions

The interactions of IECs with luminal antigens and with immune cells play a central role in determining the type of immune response triggered by intestinal microorganisms (5, 6). A critical and virtually universal early innate response of host cells to viral infection is the secretion of factors belonging to the IFN family. The secretion of IFN results in the expression of several ISGs products with antiviral activities.

We showed in different studies that the originally established porcine intestinal epithelial cell line (PIE cells) is a useful tool for studying IFN response triggered by TLR3, RIG-I, and/or MDA-5 activation. These cells are permissive to porcine RVs and also respond to dsRNA and its synthetic analog poly(I:C) (49, 50). Furthermore, co-cultures of PIE cells with immune cells isolated from porcine Peyer’s patches (PPs) provide an *in vitro* system to study the transduction of the signal from its detection by IECs to the effect on the under laying immune cells.

The response of PIE cells to poly(I:C) challenge was evaluated, and it was found that MCP-1, IL-8, TNF-α, IL-6, and both IFN-α and IFN-β were upregulated in PIE cells after stimulation (49). We also showed that after stimulation of co-cultures with poly(I:C), there was an upregulation of IFN-α, IFN-β, IFN-γ, IL-2, and IL-12p40 in immune cells (49). TLR3 was the receptor involved in the recognition of the luminal stimulus and the responsible to trigger the expression and release of cytokines, which in turn activated the underlying APCs and effector lymphocytes.

Rotavirus infection stimulates IFN-β and early antiviral gene expression by a signaling pathway that requires MAVS, an adaptor protein that is recruited to signaling complexes following activation of RIG-I or MDA-5 (51, 52). In addition, both RIG-I and MDA-5 are involved in recognizing RVs infection, as proven by the reduction of IFN-β induction when these factors are lost (51, 52). Taking into account those facts, we evaluated the suitability of PIE cells and co-cultures as models for studying this signaling pathway after RVs infection. Our results showed that PIE cells have functional TLR3, RIG-I, and MDA-5 receptors, which signal *via* IRF3 and NF-κB, inducing IFN-β and the upregulation of the ISGs MxA and RNase L (50), which are important antiviral effectors of IFN pathway.

We used PIE cells for the screening of immunobiotic LAB strains taking into consideration their ability to enhance IFN-β production upon poly(I:C) stimulation (49, 53). Thus, *L. casei* MEP221106 was selected because of its potential to impact on viral intestinal infections. *L. casei* MEP221106 had the highest capacity to improve IFN-β production in poly(I:C)-challenged PIE cells. Moreover, *in vitro* co-culture experiments showed that *L. casei* MEP221106 was able to improve not only the production of IFN-β but also the levels of other cytokines including IFN-α, TNF-α, MPC-1, and IL-6. In co-cultures of PIE cells with immune cells, we demonstrated that *L. casei* MEP221106 improved the production of inflammatory and antiviral cytokines by PPs cells when compared with control cells (49).

**Figure 4:**
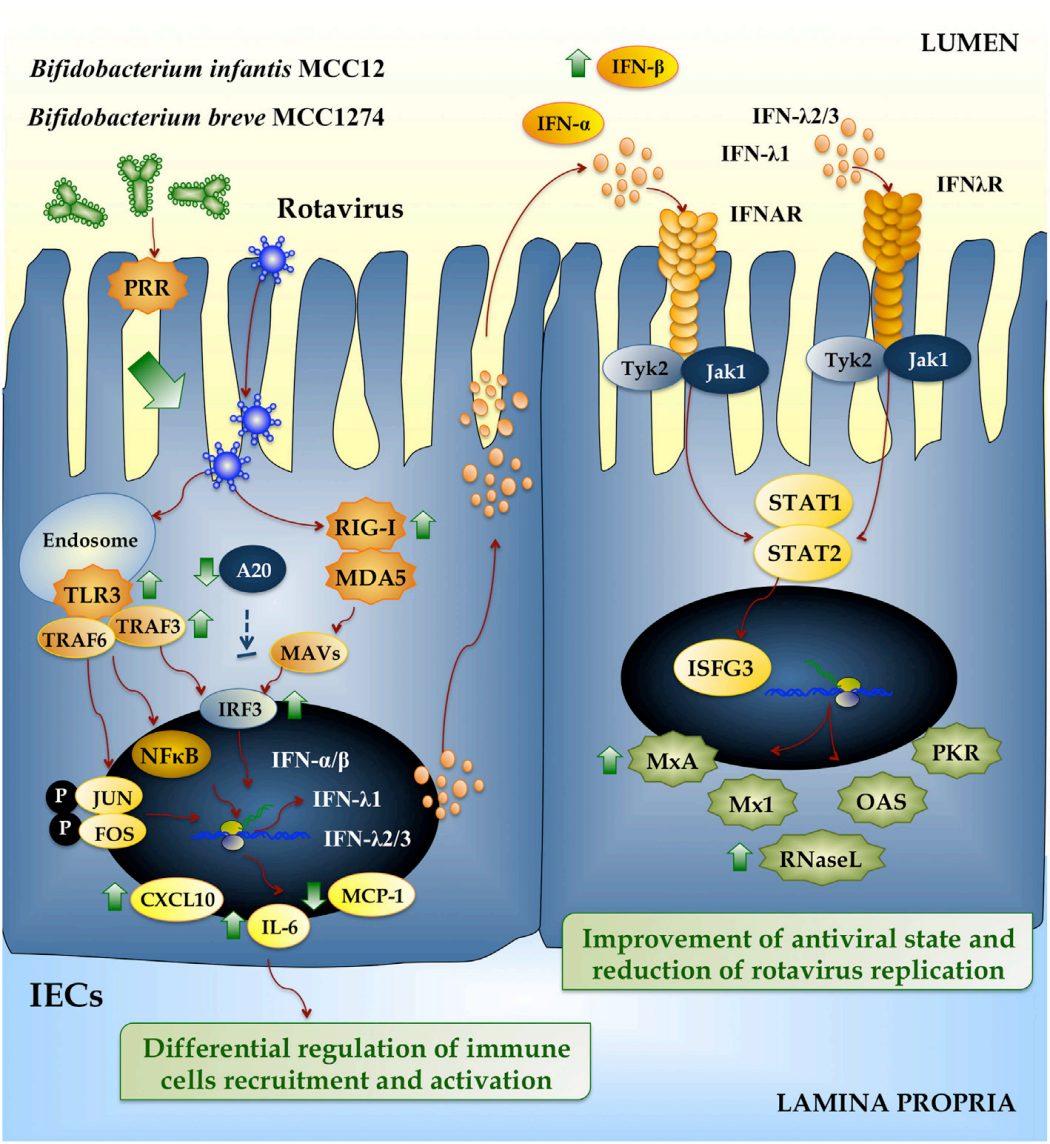
**Beneficial effects of immunobiotic bifidobacteria on the innate immune response against rotavirus in intestinal epithelial cells (IECs)**. Rotavirus double-strand genomic RNA activates toll-like receptor 3 (TLR3), retinoic acid-inducible gene-I (RIG-I), and melanoma differentiation-associated gene-5 (MDA-5), which are pattern recognition receptors (PRRs) expressed in IECs. Cellular signaling cascades mediated by interferon (IFN) regulatory factor-3 (IRF3) upregulate the expression of type I (IFN-α, IFN-β), and type III (IFNλ1, IFNλ2/3) IFN, which in turn induces the synthesis of IFN-stimulated genes with antiviral activities (MxA, Mx1, RNase L, OAS, PKR). Antiviral PRRs also activate nuclear factor κB (NF-κB) pathway and induce the secretion of proinflammatory cytokines and chemokines (IL-6, MCP-1, CXCL10). Preventive treatment of IECs with *Bifidobacterium infantis* MCC12 or *Bifidobacterium breve* MCC1274 reduce the expression of A20, increase the activation of IRF3, improve the production of the antiviral factors IFN-α, IFN-β, MxA, and RNase L, and differentially regulate the expression of IL-6, MCP-1, and CXCL10.

The PIE system was also used to screen bifidobacteria strains with anti-RVs effect (50). *Bifidobacterium infantis* MCC12 and *Bifidobacterium breve* MCC1274 were selected in the screen because they significantly upregulated IFN-β in response to poly(I:C) challenge. In addition, both MCC12 and MCC1274 strains significantly increased PIE cells resistance to RV infection (Figure 4), while other strains with moderate or no effect in IFN-β production did not have any influence on RVs replication (50). As a result of the enhanced IFN-β levels, there was a concomitant upregulation of the ISGs MxA and RNase L. These effectors of antiviral immunity have different mechanisms of action: RNase L degrades dsRNA and the resulting RNA fragments activate RLRs to amplify IFN production and induce apoptosis on virus infected cells (54), while MxA hijacks newly synthesized viral proteins into perinuclear complexes. Then, the upregulation of MxA, RNase L, and probably other ISGs induced by MCC12 and MCC1274 strains through IFN-β would be related to the lower RVs replication found in bifidobacteria-treated PIE cells. This is supported by the fact that IFN-β is a key factor for improving defenses against RVs since viral replication is restricted in permissive cells when they are pretreated with IFN-β (55). Accordingly, IFN-β treatment of newborn calves and piglets prior to RV infection reduces virus replication and disease severity (56).

Several cytokines are induced *via* NF-κB signaling as a result of RVs infection, including IL-8, RANTES, GM-CSF, GRO-α, MIP-1β, and IP-10 (57), as observed in both cell lines and histological intestinal samples. Secreted cytokines initiate an important primary line of host defense, but if this response lasts too long or is dysregulated, it may lead to tissue damage and epithelial barrier dysfunction. In this regard, we have reported that efficient regulation of inflammatory response induced by immunobiotic bacteria is essential to achieve full protection against pathogens (58, 59). In line with this, we also showed that bifidobacteria strains MCC12 and MCC1274 differentially modulated the production of proinflammatory mediators in RVs-infected PIE cells (Figure 4) (50).

Toll-like receptor negative regulators play key roles in maintaining intestinal hemostasis by regulating TLR signaling. The zinc-finger protein A20, due to its deubiquitinase and ubiquitinase E3 ligase activities, is capable to terminate TLR signaling that results in inhibition of NF-κB activation and reduction of inflammatory induced cytotoxicity (60). Saitoh et al. (61) reported that IRF3 activation is suppressed by A20. The A20 protein is able to induce the suppression of the IFN-mediated immune response and IFN-promoter-dependent transcription by physically interacting with IKK-i/IKKϵ and inhibiting dimerization of IRF3 following engagement of TLR3 by dsRNA. Moreover, A20 knock down results in enhanced IRF3-dependent transcription triggered by the stimulation of TLR3 or virus infection. Human monocyte-derived dendritic cells (DCs) stimulated with poly(I:C) upregulate A20. When A20 is downregulated in DCs, they showed higher activation of NF-κB and AP-1, which resulted in increased and sustained production of IL-6, IL-10, and IL-12p70. Furthermore, DCs enhanced their T cell stimulatory capacity (62). Negative regulators involved in TLR signaling can be modulated by immunobiotic strains in human intestinal cell lines (63). In this regard, we also reported that both *B. infantis* MCC12 and *B. breve* MCC1274 significantly reduced the expression of A20 in RVs-infected PIE cells (Figure 4) (50), which is in line with the capacity of both strains to improve IRF3 activation and IFN-β production. In line with our findings, MacPherson et al. (64) also studied the effect of probiotics in the modulation of poly(I:C) induced inflammatory response in HT-29 cells. Stimulating HT29 cells with poly(I:C) alone increased the expression of A20, but the co-stimulation with poly(I:C) and probiotics significantly reduced A20 expression levels.

**Figure 5:**
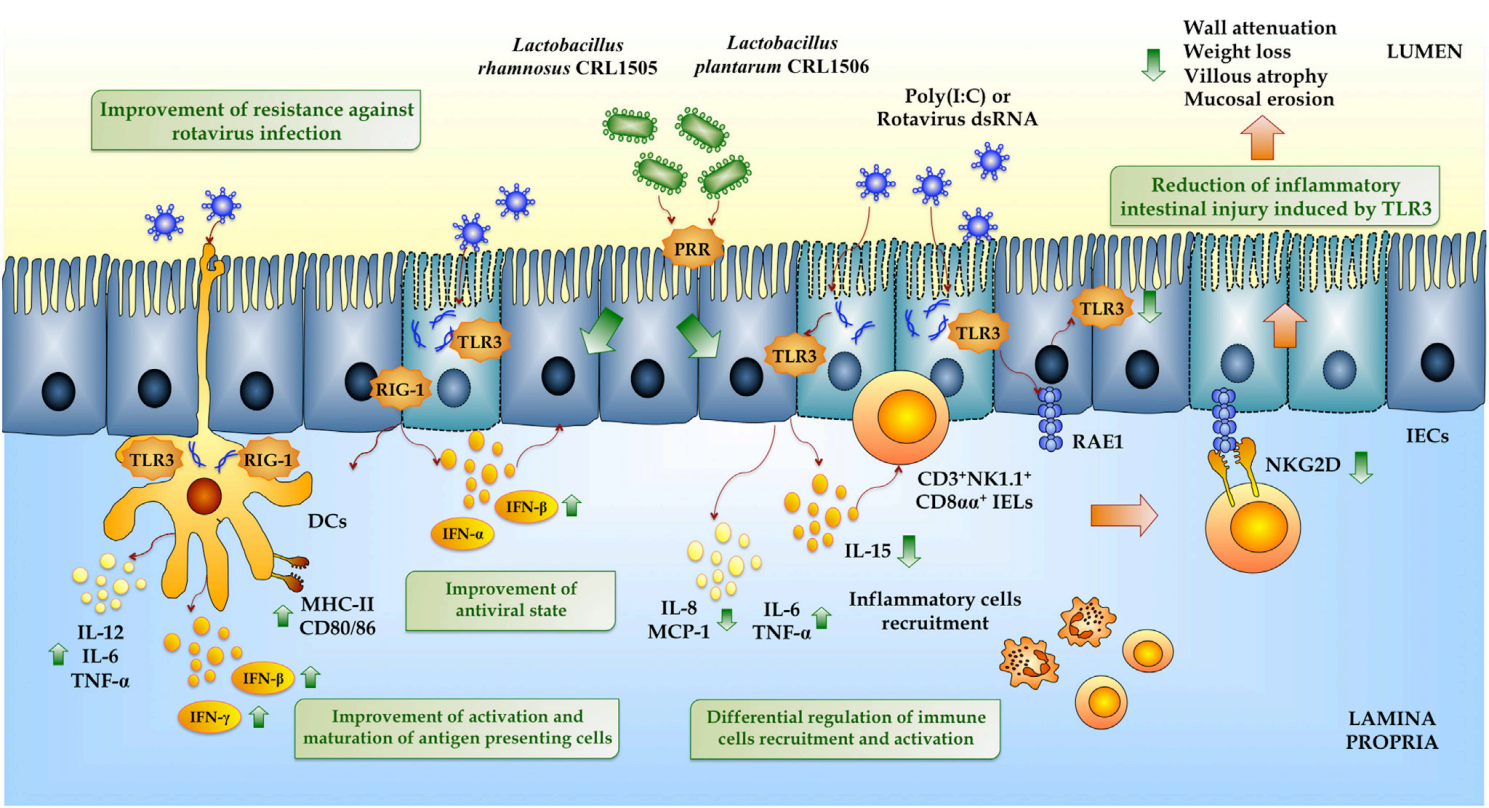
**Beneficial effects of immunobiotic lactobacilli on the innate immune response against rotavirus in intestinal mucosa**. Rotavirus double-strand genomic RNA or poly(I:C) activate toll-like receptor 3 (TLR3), retinoic acid-inducible gene-I (RIG-I), and melanoma differentiation-associated gene-5 (MDA-5), which are pattern recognition receptors (PRRs) expressed in intestinal epithelial cells (IECs) and dendritic cells (DCs). Activation of antiviral PRRs increases the production of IFN-α, IFN-β, IFN-γ, and proinflammatory cytokines and chemokines (TNF-α, IL-6, IL-8, IL-12, MCP-1), which improves the antiviral state in IECs, induces the recruitment and activation of immune cells and the maturation of DCs. In addition, both purified rotavirus genomic dsRNA and poly(I:C) activate TLR3 in IECs increasing the expression of IL-15 and retinoic acid early inducible-1 (RAE1). IL-15 produced by IECs induces the recruitment of CD3^+^NK1.1^+^CD8αα^+^ intraepithelial lymphocytes (IELs), which mediates epithelial destruction and mucosal injury by the NKG2D receptor expressed on these cells that is able to recognize RAE1. Preventive treatments with *Lactobacillus rhamnosus* CRL1505 or *Lactobacillus plantarum* CRL1506 improve the production of type I IFN and IFN-γ in the intestinal mucosa enhancing the antiviral state and differentially regulate the expression of inflammatory cytokines and chemokines reducing the intestinal damage, especially associated with the TLR3–IECs–IELs interaction.

We also used these porcine *in vitro* systems to attain deeper knowledge into the mechanisms involved in the immunomodulatory effect of *L. rhamnosus* CRL1505 and concentrated our attention in the crosstalk between the immunobiotic strain and porcine IECs and APCs, in order to explain its capacity to reduce viral diarrhea episodes in children (44). Moreover, we performed comparative studies with another immunobiotic strain, *Lactobacillus plantarum* CRL1506, that is able stimulate intestinal immunity in animal models (65). Studies comparing the immunobiotic strains *L. rhamnosus* CRL1505 and *L. plantarum* CRL1506 in co-cultures of PIE cells and APCs, stimulated with poly(I:C), showed that both strains improved the production of type I IFNs in response to poly(I:C) challenge (66). In addition, CRL1505 and CRL1506 strains modulated the expression of proinflammatory and regulatory cytokines and influenced activation and maturation of APCs (Figure 5). However, *L. rhamnosus* CRL1505 had a stronger effect both when applied alone or combined with a posterior poly(I:C) challenge. The improved Th1 response induced by immunobiotic lactobacilli was evidenced by the augmented expression of MHC-II, IL-1β, IL-6, and IFN-γ in DCs (66, 67). Those studies gave scientific basis for explaining the protection against intestinal viral infections achieved by *L. rhamnosus* CRL1505 in children.

The receptors, which are activated by the immunobiotics strains with antiviral capabilities MEP221106, MCC12, MCC1274, CRL1505, and CRL1506 strains in PIE cells to reduce A20, improve IRF-3 activation and increase IFN-β production remains to be uncovered. Bifidobacteria strains with a high capacity to stimulate TLR2 such as *B. longum* BB536 and *B. breve* M-16V were able to increase the expression of A20 in PIE cells and reduce TLR4-mediated inflammatory response (68, 69). On the contrary, strains with low capacity of stimulating TLR2 did not modify the expression of the ubiquitin-editing enzyme A20 in PIE cells challenged with TLR4 agonists. In our experiments, we were unable to block the increase of IFN-β induced by the lactobacilli and bifidobacteria by using anti-TLR2 or anti-TLR9 antibodies, suggesting that other receptor(s) are involved in the immunobiotic activity (66). Further studies are needed in order to find the PRRs involved in the recognition of lactobacilli and bifidobacteria leading to A20 and IFN-β modulation in PIE cells.

Recently, we confirmed *in vivo* the differential antiviral immunomodulatory activities triggered by *L. rhamnosus* CRL1505 and *L. plantarum* CRL1506 (65). Both strains increased the production of IFNs, the CRL1505 treatment being the most effective for increasing the levels of IFN-γ. Then, our results suggest that these two lactobacilli strains have potential to be used to improve the outcome of viral gastrointestinal disease. This is also supported by the human clinical trial demonstrating the capacity of *L. rhamnosus* CRL1505 to improve the infectious disease rates in children (70). Recently, Zhang et al. (71) proposed the activation of innate immunity with flagellin as a preventive and therapeutic strategy against RVs infection. They demonstrated that intraperitoneal flagellin injection reduced severity and shedding of RVs RNA in acute and chronic infected mice *via* TLR5/NLRC4 activation, which resulted in secretion of IL-22 and IL-18 by different effector cells. Although the mechanism of action of flagellin administration is substantially different to the mechanisms elicited by immunobiotics, both approaches rely on the principle of combating viral infection by enhancing innate immune defenses.

*Lactobacillus rhamnosus* CRL1505 and *L. plantarum* CRL1506 also reduced TLR3-induced small intestinal injury by regulation of proinflammatory cytokines production and IECs–IELs interaction (65) (Figure 5). IECs and IELs are the first line of defense against pathogens including viruses, and their interaction is essential for maintaining an appropriate immunological homeostasis. IECs produce a variety of cytokines and chemokines, including IL-6, IL-7, IL-8, IL-15, TNF-α, TGF-β, and GM-CSF. IL-15 functions as a mediator of TLR3-induced small intestinal injury (24). Abnormal TLR3 signaling results in elevated levels of IL-15, which regulates IECs apoptosis by activating perforin-mediated killing by CD3^+^NK1.1^+^ IELs (70). Moreover, IL-15 is able to enhance the cytotoxic activity of human IELs (72). Blocking the α receptor of IL-15 partially protected mice from poly(I:C)-induced small intestinal injury, including less villous atrophy, and mucosal erosion (24). Autologous ligands released by cells stress and infection are recognized by the NKG2D receptor in NK cells. NKG2D ligands expression is downregulated by gut microbiota, as demonstrated in germ-free mice, which had increased surface expression of these ligands (73). RAE1, a high affinity NKG2D ligand, which is minimally detected in normal cells, is upregulated upon TLR3 activation. In fact, blockade of NKG2D–RAE1 interaction avoids the cytotoxic effect of IELs on IECs and prevents acute small intestinal injury in mice challenged with dsRNA (27). Therefore, TLR3 signaling stimulates IECs to express IL-15 and RAE1 and induces CD3^+^NK1.1^+^CD8αα^+^ IELs to express NKG2D through IEC-derived IL-15. In our hands, poly(I:C) treatment of mice increased intestinal injury in a IL-15- and CD8αα^+^NKG2D^+^-dependent manner (65). Poly(I:C) induced inflammatory-mediated intestinal tissue damage through the increase of CD3^+^NK1.1^+^ and CD8αα^+^NKG2D^+^ cells as well as proinflammatory mediators (TNF-α, IL-1β, IFN-γ, IL-15, RAE1, IL-8). Mice pretreated with immunobiotic lactobacilli before TLR3 activation responded with reduced levels of TNF-α, IL-15, RAE1, CD3^+^NK1.1^+^, CD3^+^CD8αα^+^, and CD8αα^+^NKG2D^+^ cells (Figure 5). The beneficial effect of these lactobacilli improved mice health as reflected by a significant reduction of body weight loss and intestinal tissue damage after poly(I:C) challenge (65).

It is well known that commensal bacteria in the gut are able to modulate IELs function. Furthermore, IELs are significantly reduced in germ-free mice (74, 75) underlying gut microbiota importance in the maintenance of IELs. These specialized lymphocytes are very important players in mucosal protection; they seem to occupy a unique temporal niche from which they are able to detect and limit bacterial penetration already in the first hours after pathogen attack (76). Ismail et al. (76) showed that IEL antibacterial response depends on bacterial stimulation in a MyD88-dependent signaling. Later, Jiang et al. (77) investigated the role of NOD2 signaling in the maintenance of IELs and found that NOD2 maintained IELs *via* recognition of gut microbiota. They demonstrated that stimulation of IEL requires activation of PRRs signaling in neighboring IECs (76, 77).

Therefore, it was shown that commensal bacteria establish a regulatory milieu in a healthy gut, with increased expression of immuno-inhibitory cytokines such as TGF-β and IL-10, which in turn downregulate NKG2D ligand surface expression (78, 79). This is in line with our findings for the immunomodulatory strains *L. rhamnosus* CRL1505 and *L. plantarum* CRL1506, which reduced expression of RAE-1 and increased levels of intestinal IL-10. Whether the immunomodulatory effects of *L. rhamnosus* CRL1505 and *L. plantarum* CRL1506 are induced by direct action on the IECs (indirectly on IELs) and/or a direct effect on IELs is an open question, which we propose to address in the near future.

## Conclusion

The detailed characterization of the cellular and molecular mechanisms underlying the intestinal innate defense against RV infection achieved in the past years has opened new ways for developing strategies to preventing and treating this viral induced diarrhea. In this sense, the use of immunobiotic bacteria to beneficially modulate IFN and inflammatory signaling pathways in IECs and immune cells is an attractive target for preventive or therapeutic intervention against RVs infection. Furthermore, the advances in the knowledge of the molecular crosstalk between immunobiotics and the gut innate immune system have provided light into the microorganism-sensing signals that allow these beneficial microorganisms to improve intestinal immune responses. This new molecular information might be helpful to improve the development of functional foods and/or pharmabiotics using immunobiotics aimed to reduce mortality and severity of RVs disease.

## Author Contributions

JV, MV-P, and HK designed, wrote, and revised the review article.

## Conflict of Interest Statement

The authors declare that the research was conducted in the absence of any commercial or financial relationships that could be construed as a potential conflict of interest.
